# Camera-LiDAR Multi-Level Sensor Fusion for Target Detection at the Network Edge

**DOI:** 10.3390/s21123992

**Published:** 2021-06-09

**Authors:** Javier Mendez, Miguel Molina, Noel Rodriguez, Manuel P. Cuellar, Diego P. Morales

**Affiliations:** 1 Infineon Technologies AG, Am Campeon 1-15, 85579 Neubiberg, Germany; Miguel.MolinaFernandez2@infineon.com; 2 Department of Electronic and Computer Technology, University of Granada, Avenida de Fuente Nueva s/n, 18071 Granada, Spain; noel@ugr.es (N.R.); diegopm@ugr.es (D.P.M.); 3 Department of Computer Science and AI, University of Granada, C/Periodista Daniel Saucedo Aranda s/n, 18071 Granada, Spain; mpcuellar@ugr.es

**Keywords:** sensor fusion, deep learning, edge computing, camera sensor, LiDAR sensor, target detection

## Abstract

There have been significant advances regarding target detection in the autonomous vehicle context. To develop more robust systems that can overcome weather hazards as well as sensor problems, the sensor fusion approach is taking the lead in this context. Laser Imaging Detection and Ranging (LiDAR) and camera sensors are two of the most used sensors for this task since they can accurately provide important features such as target´s depth and shape. However, most of the current state-of-the-art target detection algorithms for autonomous cars do not take into consideration the hardware limitations of the vehicle such as the reduced computing power in comparison with Cloud servers as well as the reduced latency. In this work, we propose Edge Computing Tensor Processing Unit (TPU) devices as hardware support due to their computing capabilities for machine learning algorithms as well as their reduced power consumption. We developed an accurate and small target detection model for these devices. Our proposed Multi-Level Sensor Fusion model has been optimized for the network edge, specifically for the Google Coral TPU. As a result, high accuracy results are obtained while reducing the memory consumption as well as the latency of the system using the challenging KITTI dataset.

## 1. Introduction

The interest in autonomous vehicles has increased in recent years due to the advances in multiple engineering fields such as machine learning, robotic systems and sensor fusion [1]. The progress of these techniques leads to more robust and trustworthy computer vision algorithms. Using sensors such as Laser Imaging Detection and Ranging (LiDAR), radar, camera or ultrasonic sensors with these techniques enables the system to detect relevant targets in highly dynamic surrounding scenarios. These targets may include pedestrians, cyclists, cars or motorbikes among others, as discussed in public autonomous car datasets [2,3].

Computer vision algorithms such as You Only Look Once (YOLO) [4], Region based Convolutional Neural Network (R-CNN) [5], Fast R-CNN [6] or Single Shot Detector (SSD) [7] have been designed upon the previously mentioned sensors leading to numerous models for target detection. These models calculate the score of the bounding box location for each detected target as well as its classification based on high-level features generated by using Convolutional Neural Networks (CNN). Nevertheless, these models are not robust since the use of only one sensor may result in target detection problems in hazard situations. There are machine learning (ML) attacks that can affect single sensor models such as models only based on camera data. These attacks slightly modify target data in a scene to get a different classification result [8,9,10]. In addition, sensor data information can be degraded due to weather conditions [11,12,13]. Other sensors can also be attacked with similar results, as is the case of the LiDAR [14,15] or radar [16,17].

One viable option to improve the reliability of these systems as well as to improve the accuracy of the results is the sensor fusion. This technique implements a system with multiple data sources to complement the inputs. This approach results in a more complete knowledge of the scenario for a better computer vision. The sensor fusion approach can be divided into multiple techniques: *Early Fusion*, *Late Fusion* and *Intermediate Fusion* [18]. In the *Early Fusion* the raw data or low-level preprocessed data is combined to generate a more complete raw data while in *Late Fusion* high-level features such as target location are merged for a better final result. *Intermediate Fusion* can be understood as a combination of both previous techniques in which the data is merged at multiple levels to effectively find the merged representation of multiple input data [19]. Recently, some authors have proposed computer vision models based on these approaches [11,19,20].

Most of these studies focus on improving the detection algorithms without taking into account the constraints imposed by the autonomous vehicle industry of latency and privacy to ensure the safety of the passengers [1]. At the same time, the limitations of the final system in which the models should be integrated must be studied to ensure the suitability of the models for the system regarding memory and computing power requirements due to the specifications of the processing units deployed at the network edge [21,22]. Because of this, in our paper we focused on researching a sensor fusion algorithm that can be deployed in edge devices. These devices are meant to work without a high frequency communication with external devices to process the data at the network edge. One of the advantages of this is the increase of the security of the raw data since it is not broadcast to an external device. However, they have constraints regarding memory in the device and computing power due to their size and energy consumption.

Our proposed algorithm is based on the fusion of LiDAR and camera data. LiDAR data provides reliable information of depth and target presence which can complement the high-quality camera image data of the surroundings for the target classification. Consequently, the LiDAR provides further information about the relevant areas of the camera image. The combination of these sensors can be used to ensure the presence of the targets and to provide a solution for scenarios where one of the sensors does not provide data. Our algorithm, called Multi-Level Sensor Fusion (MLSF), executes the data fusion at multiple levels using the *Intermediate Fusion* as a bidirectional reinforcement approach for both input data. A new layer structure, *fusion layer*, has been integrated in the proposed deep learning model. This layer generates a shared feature map which is later used as a mask for the feature map of the LiDAR and camera to further extract relevant features before the final target localization and classification. Because of this, the target detection results significantly improve. The latency and memory consumption have been used as constraints during the design of the model to ensure its suitability for the network edge, specifically the Google Coral TPU edge device.

This model has been evaluated using the challenging KITTI dataset [2], where it achieves a final latency of 0.057 s and accuracy of 90.92%, 87.81% and 79.63% for easy, medium and hard to detect targets.

The manuscript is organized as follows: Section 2 focuses on related works to the addressed problem. In Section 3, the proposed Deep Fusion Network is described for a deep understanding of its structure as well as the used input data. After that, Section 4 will focus on the experiment where this algorithm has been applied, and finally Section 5 is the conclusion.

## 2. Related Works

Since autonomous vehicles are a current trend, numerous authors are researching the sensor fusion applied to this topic in order to improve the state-of-the-art accuracy results. As previously mentioned, some of them research single-sensor scenarios for target detection in the autonomous vehicle paradigm such as Deep Manta [23]. This model uses only the data from a monocular camera to generate 2D and 3D bounding boxes as well as classification for targets. At the same time, this model provides information regarding the visibility of each of the targets’ parts, making it suitable for annotation tasks. However, due to its numerous algorithms to extract the location information of each part of the target, its visibility and classification, this model has high execution times in comparison with other techniques such as our proposed model.

Nevertheless, numerous authors are starting to research the use of LiDAR sensors for mapping and detection due to the accuracy of this sensor to generate point clouds based on the surfaces in the environment. This sensor has been applied to other research topics apart from autonomous vehicles; it is used in the agricultural industry to generate accurate maps. M.P. Christiansen et al. [24] developed a UAV Mapping System for Agricultural Field Surveying where LiDAR data was gathered using an Unmanned Aerial Vehicle (UAV) device. Global Navigation Satellite System (GNSS) and Inertial Measurement Unit (IMU) sensors were also integrated in the device in order for the point cloud reconstruction based on multiple frames to be later used for tasks such as estimation of the soil surface and total plant volume. Following the same research line, A. Patil et al. [25] proposed a framework to align LiDAR and video data using a point cloud registration algorithm. By using this framework in five different experiments, they proved how it can help to reduce the time to complete retrofitting tasks by 20% on average.

LiDAR sensors have also been used for other topics such as autonomous cars. One of the authors who researched this topic is J.Zarzar. This author proposed a target detection algorithm, PointRGCN, based on a single LiDAR sensor [26]. This algorithm is based on Graph Convolutional Networks integrated in a multiple 3D object detection pipeline. By doing so, the bounding boxes can be refined multiple times to achieve state-of-the-art accuracy results. Nevertheless, even if the model provides high accuracy results, it faces the same problems previously stated with the Deep Manta model regarding adversarial attacks or lack of information.

Similar to our proposed algorithm, other researchers are studying the implementation of a sensor fusion technique for target detection. One of the proposed algorithms following this research line is the Camera-LiDAR Object Candidates Fusion (CLOCs) Deep Neural Network (DNN) architecture proposed by S. Pang et al. [27]. This network combines LiDAR and camera data to locate and classify targets in 2D and 3D. Depending on the desired target detection, 2D or 3D, the system uses different perspectives obtained from the raw LiDAR data. As a result, this technique provides high accuracy location and classification of the targets in 2D and 3D. However, due to the complexity of its detectors, its memory consumption may not be suitable for the current edge devices.

Following the same research line for target detection using sensor fusion, J. Kim et al. [19] proposed a DNN to combine LiDAR depth maps and camera images. Its approach is based on extracting relevant features from both sensor data independently using the VGG-16 structure [28] before fusing the output feature maps at multiple levels. This approach is similar to our proposed network; however, we included the option of ignoring the result of the previous fusion layers to avoid including not highly relevant data in the final data fusion step. This leads to a more efficient approach where only relevant information is further studied. At the same time, this model presents the same problem regarding the network edge as the CLOCS Deep Neural Network. Due to the detector implemented as well as the structure of the layer used for the data fusion, the latency and memory consumption are larger than that obtained with our model.

LiDAR and camera are not the only sensors researched for target detection in the vehicle context. Other authors research techniques based on different devices such as camera, radar or ultrasonic sensors due to the high cost of the LiDAR sensors. Because of this, F. Nobis et al. [11] researched the fusion of radar and camera data for this task. In this pipeline, the radar data is preprocessed to generate 2D coordinates in the horizontal plane which could belong to possible targets. Their approach was based on DNN where the data is fused on multiple levels. During the training phase of the model, the weight configuration of the DNN establishes at what level the fusion is more effective to obtain the desired output. The accuracy achieved with this pipeline in the NuScenes [3] dataset is 55.99%, requiring 56 ms to study each frame.

Therefore, it can be observed how multiple approaches for the target detection are being researched and provide high accuracy results in some of the most popular datasets such as KITTI or NuScenes. In the case of single-sensor models, the problem of adversarial attacks or problems during the data acquisition are not solved, as [11] explains. This is one of the most relevant reasons to use a sensor fusion approach as other previously mentioned authors have done [11,19,27]. However, none of these models take into account the constraints of the autonomous driving industry regarding latency and memory consumption. Because of this, our research faces the problem of the target detection from the Edge Computing perspective. The model integrates detectors that have been proven to provide state-of-the-art results at the network edge while also optimizing the model at layer and network level. Therefore, our proposed model considers the constraints of the autonomous vehicles paradigm while maintaining state-of-the-art accuracy results. A deeper comparison of the mentioned techniques, as our model, is shown in Section 4.

## 3. Proposed Multi-Level Sensor Fusion Network

The proposed Multi-Level Sensor Fusion (MLSF) Network aims to detect targets in the camera data by integrating LiDAR data as reinforcement data. Camera and LiDAR data are fused through this network at multiple levels, enabling the system to merge the features at the specific level or levels decided during the training phase of the model. The SSD [7] structure has been used as a reference for the proposed network due to its reduced memory consumption while maintaining high-performance accuracy results.

In order to optimize the process as well as the memory consumption, the LiDAR data preprocessing has been studied to reduce its dimensions while maintaining most of its relevant features for the object detection before it is fed into our proposed model.

### 3.1. LiDAR Depth Map Representation

A Laser Imaging Detection and Ranging (LiDAR) sensor has been used in this project to gather information regarding the environment. Differently from the camera, the LiDAR sensor transmits laser pulses and measures the time it takes until a reflection is received. Based on this, it calculates the distance of the target and its 3D coordinates since the angles used to send the laser pulse and the distance are known. Consequently, this sensor provides information regarding surfaces rather than only images as the camera does.

The order of these 3D points, *(x, y, z)*, in datasets such as KITTI [2] and NuScenes [3] public datasets cannot be ensured since it depends on the sensor used to gather data as well as the environment. Therefore, it is usually assumed that the order of the points is unknown, unlike pixel arrays in images. In consequence, their integration in DNNs is not straightforward since the network must be invariant to these permutations of the input data in the feeding order.

**Figure 1:**
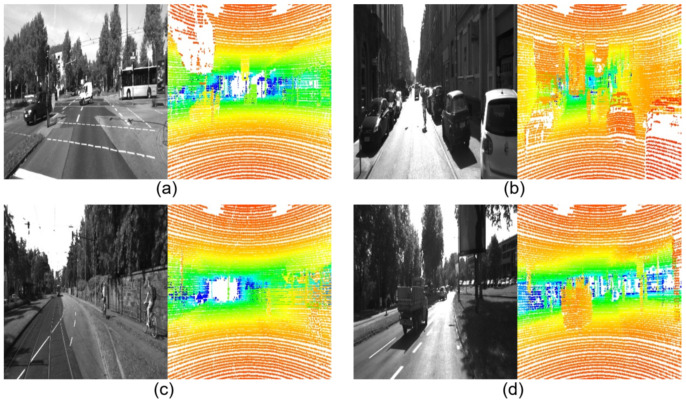
Camera images (left image in (**a**–**d**)) and LiDAR depth maps generated from LiDAR raw data (right image in (**a**–**d**)).

To overcome this challenge, the voxel grid approach is applied by numerous authors by using 3D-CNN [20,29]. However, this technique increases the complexity of the DNN, leading to larger models, and uses layer structures that are not supported by some edge devices such as the 3D convolutional layer. Therefore, rather than this technique, depth maps from LiDAR data have been generated as shown in Figure 1. This technique ensures the low memory consumption and latency of the model in comparison with the voxel approach.

Depth maps representations reduce the dimensions of the LiDAR data to generate 2D images. The original 3D points are codified to generate these images following (1)–(4) procedures for each point. These equations transform the 3D points from Cartesian coordinates to the new representation system.

(1) $$x_{DepthMap}=tan^{-1}\left(y_{3D}/x_{3D}\right)$$

(2) $$d_{3D}=\sqrt{x_{3D}^{2}+y_{3D}^{2}+z_{3D}^{2}}$$

(3) $$y_{DepthMap}=cos^{-1}\left(z_{3D}/d_{3D}\right)$$

(4) $$color_{DepthMap}=d_{3D}$$

These new coordinates can be directly used for the representation of the depth maps by adding the color component. Since the new data type is a 2D image, the SSD structure can be implemented for the feature extraction from LiDAR depth maps. This preprocessing technique is applied to the raw LiDAR data before being fed into our proposed MLSF model.

This preprocessing of the LiDAR data enables a memory consumption reduction of 95.6% to store the data when the depth maps are saved as 300 × 300 pixel images in comparison with the raw 3D point cloud.

### 3.2. Overall System Description

**Figure 2:**
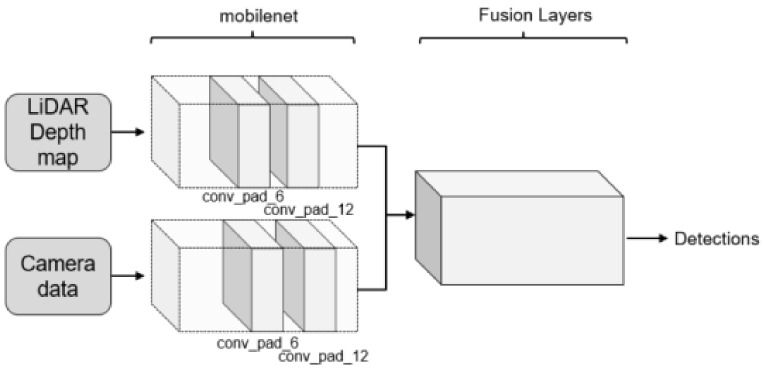
Proposed Multi-Level Sensor Fusion network structure for target detection.

**Figure 3:**
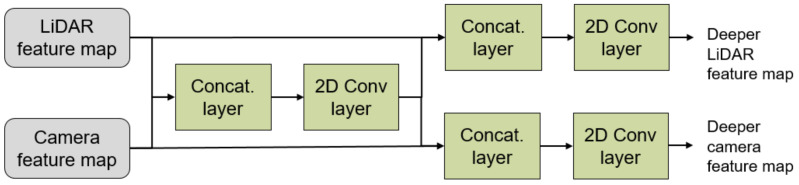
Proposed fusion layer.

The structure of the proposed Multi-Level Sensor Fusion network is shown in Figure 2. The camera images as well as the LiDAR depth maps are fed separately to our MLSF model. The two input data are studied using two separated CNNs following the SSD structure to generate the feature maps from each data type. These CNNs use the mobilenet network backbone. As a result of this, the initial number of layers is smaller than other structures such as VGGNet-16 used by other authors. The fusion of the feature maps is executed by our *fusion layers* shown in Figure 3.

The *fusion layers* combine the feature maps from both CNNs by concatenating them (in the channel axis) before applying a 2D convolutional filter (3 × 3) with ReLU activation function. After this, the new feature map is concatenated individually to each of the previous initial feature maps from the LiDAR and camera data to generate deeper feature maps. To maintain the shape of the initial feature map as well as to further process the data, another 2D convolutional filter (1 × 1) with ReLU activation function is applied to each of the new deeper feature maps. These feature maps can be used in a later step as input for another fusion layer, enabling the system to execute a multi-level fusion of the data. Because of this structure, the network learns during the training phase at what level it must execute the fusion of each feature extracted from the LiDAR and camera sensors.

In our network, four of these *fusion layers* have been implemented in order to extract the information of the localization of the targets and another four independent *fusion layers* for the classification. The initial feature maps for the classification are extracted from the *conv-pad-6* layer of the mobilenet networks that process the camera and LiDAR data. For the localization, the initial feature maps are extracted from the *conv-pad-12* layer in each network.

Since our approach is based on the SSD structure, the loss function implemented in the system is ruled by the SSD loss presented in (5)–(14).

(5) $$L_{loc}\left(x,l,g\right)=\sum_{i\in Pos}^{N}\sum_{m\in (cx,cy,w,h)}x_{ij}^{k}\times S_{L1}$$

(6) $$S_{L1}=smootth_{L1}\left(l_{i}^{m}-{\hat{g}}_{j}^{m}\right)$$

(7) $$smooth_{L1}\left(l_{i}^{m}-{\hat{g}}_{j}^{m}\right)$$

(8) $${\hat{g}}_{j}^{cx}=\left(g_{j}^{cx}-d_{i}^{cx}\right)/d_{i}^{w}$$

(9) $${\hat{g}}_{j}^{cy}=\left(g_{j}^{cy}-d_{i}^{cy}\right)/d_{i}^{h}$$

(10) $${\hat{g}}_{j}^{w}=log\left(\frac{g_{j}^{w}}{d_{i}^{w}}\right)$$

(11) $${\hat{g}}_{j}^{h}=log\left(\frac{g_{j}^{h}}{d_{i}^{h}}\right)$$

(12) $$L_{conf}\left(x,c\right)=-\sum_{i\in Pos}^{N}x_{ij}^{p}log({\hat{c}}_{i}^{p}-\sum_{i\in Neg}log\left({\hat{c}}_{i}^{0}\right)$$

(13) $${\hat{c}}_{i}^{p}=\frac{exp(c_{i}^{p})}{\sum_{p}exp\left(c_{i}^{p}\right)}$$

(14) $$L\left(x,l,g\right)=\frac{1}{N}\left(L_{conf}\left(x,c\right)+\alpha L_{loc}\left(x,l,g\right)\right)$$

where *N* indicates the number of matching default boxes, *l* represents the predicted boxes, *g* the ground truth boxes, *x* the coordinates of the bounding boxes and *c* the class confidences. These parameters also include the offset for the center points (*cx*, *cy*), the width of the box (*w*) and its height (*h*).

This DNN has later been optimized for the network edge by pruning the layers. This process removes the connections when the parameters of a layers/neurons do not highly modify the value of the input signal, consequently reducing the size of the model and the number of operations. At the same time, due to the constraints of the Google Coral TPU Edge Device where the model should be integrated, the model parameters have been quantized to 8-bit integer values. After the optimization process, the final model requires 56.4 MB in contrast to the initial model with a size of 185 MB.

**Figure 4:**
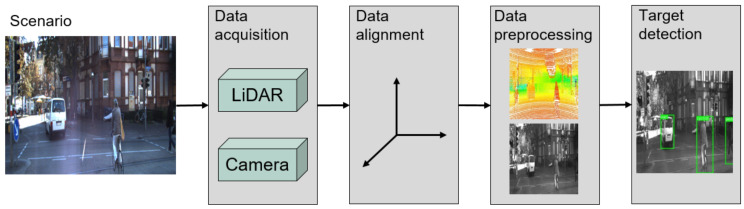
Full pipeline for target detection using our proposed model.

Following these techniques, the full pipeline from the data acquisition to target detection is shown in Figure 4. In the first moment, after gathering the data from the LiDAR and camera sensors, the data need to be aligned to ensure that the coordinate origin is shared by both sensors. This is also used to filter out parts of the scene visualized only by the LiDAR that are not relevant for the target detection. After this step, the LiDAR raw data is used to generate the LiDAR depth maps previously explained. The input data also needs to be quantized to 8-bit integers before it is fed into the model due to the constraints imposed by the Google Coral TPU Development Board. Finally, our proposed MLSF model studies the input data to provide information about the targets in the current frame.

## 4. Experiment

In this section, our proposed MLSF model is evaluated using the KITTI dataset [2] to compare its accuracy results for the 2D target detection with state-of-the-art techniques. At the same time, latency and memory size are also included in the comparison since the goal is to design a target detection model for the network edge. Furthermore, the influence of the lighting conditions will also be discussed in this section.

**Figure 5:**
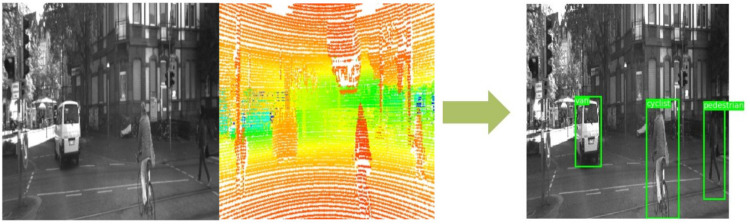
Input data on the left side of the figure and output on the right side.

Since targets could be located in both sensor data, the coordinates’ origin has been set to the ego vehicle for an easier final evaluation of the model results, as depicted in Figure 5.

### 4.1. Dataset

The dataset used for this experiment is the KITTI dataset [2] since it is one of the most popular databases when LiDAR and camera data is required. Due to this, numerous state-of-the-art results provide accuracy information of their algorithms with this dataset. The LiDAR sensor used in this dataset is the Velodyne HDL-64E [30] developed by Velodyne in San Jose, United States.

This dataset consists of 7481 training samples and 7518 testing samples. Both subsets include camera and LiDAR data. The labels in this dataset are provided using the ego vehicle coordinate system that can be used for all the sensors integrated in the KITTI dataset. Since our goal is the 2D target detection in the camera images, we have converted the LiDAR and camera data to this ego vehicle coordinate system in order to match the labels with the used data. The labeled targets in this dataset are: car, pedestrian, bicycle, tram, van, truck, misc and sitting down person. These targets’ labels also include information about the difficulty to detect the target (easy, medium and hard). Therefore, we will also provide the accuracy result achieved on each of these subsets based on the difficulty of the targets.

Since labels are required to measure the final accuracy of the model, the training dataset has been split into training and evaluation data using 30% of the dataset for the evaluation.

### 4.2. Hardware Architecture

**Figure 6:**
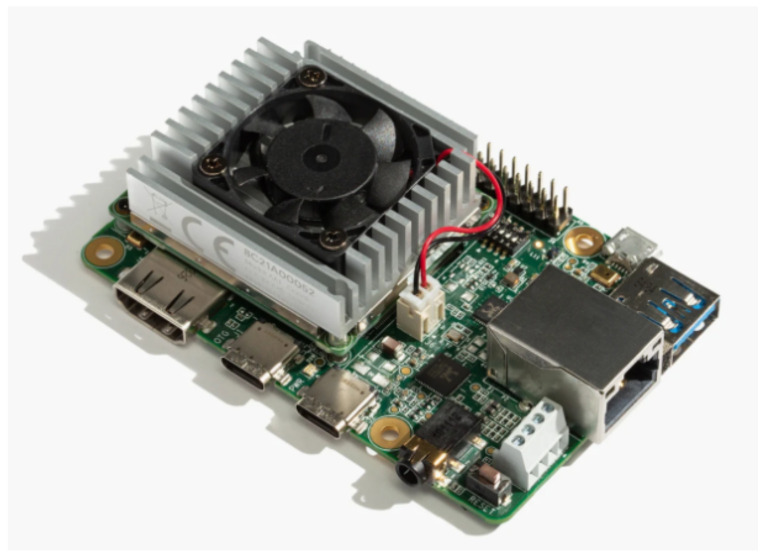
Google Coral TPU Development Board image from https://coral.ai/ (accessed on 31 May 2021) [31].

As the goal of this paper is to present a sensor fusion pipeline for target detection at the network edge, the latency results were calculated in an edge device. The specific device is the Google Coral TPU Dev Board from Google, manufactured in China, that is shown in Figure 6. This platform is based on the integration of a TPU coprocessor to execute the tensor computations in a more efficient way than using traditional CPUs or GPUs. However, this device still integrates an Integrated GC7000 Lite Graphics GPU and an NXP i.MX 8M SoC CPU to execute nontensor operations. As a result of this, it is capable of performing 4 trillion operations per second (TOPS) while only consuming 0.5 W/TOPS.

Simultaneously, the software framework for the implementation of DNNs in the device enables the optimization of them. As a result, the latency, as well as the size of the model, can be reduced in most cases.

In order to provide an accurate comparison of the latency, the compared algorithms have been implemented in the same platform when possible. In other cases, the results of the algorithms have been extracted from the original papers and KITTI dataset results table [2].

### 4.3. Experimental Settings

The parameters used during the training of the model can be observed in Table 1. The loss function used for the training of the model is the same as that used in the SSD structure, which is explained in Section 3.2.

The number of labels for each of the classes of the KITTI dataset is not the same, having large variation between common classes such as car and uncommon classes such as tram. Therefore, the algorithm for the model training tries to use the same number of labels from each of the studied classes to overcome this class imbalance problem while still using all the training data.

The Intersection Over Union (IOU) algorithm has been implemented to measure the accuracy of the model. This technique can be applied to any system that predicts bounding boxes in scenarios where the ground truth is known. An IOU result above 0.5, as used in this experiment for Pedestrian and Cyclist classes, is normally considered as a good prediction. For cars, the IOU threshold selected is 0.7 as generally done when using the KITTI dataset. The algorithm itself is explained in (15), where A means the ground truth bounding box and B the predicted bounding box.

(15) $$IOU=\frac{A\cap B}{A\cup B}$$

As well as the configuration of the experiment, it is also important to comment on the algorithms that have been selected for the comparison with our model. Due to the fact that the main goal of our research is the target detection at the network edge using sensor fusion, we have selected some of the most relevant target detection algorithms which have been tested using the KIITI dataset. Among those algorithms, we provide a comparison with the models based on LiDAR and camera sensor fusion. As a result, a comparison of models which share the dataset as well as the application goal is shown in the next section. These models are the Deep Gated Information Fusion Network (DGFN) [19], CLOCs [27], Multi-view 3d object detection network (MV3D) [32] and Multi-Scale Convolutional Neural Network (MS-CNN) [33] since they are the most relevant in this topic to the best of our knowledge.

At the same time, a comparison with single sensor models is also discussed in the next section. The goal of this comparison is the discussion of the advantages of the sensor fusion applied for the target detection rather than using a single sensor. The models used for this comparison are the SSD (studied separately to detect targets using LiDAR and camera data) [7], the Deep Manta [23], the Structure Aware SSD (SA-SSD) [34] and the MonoPair [35] algorithms. These models have been chosen because SSD is one of the most efficient model structures for the network edge. The other models (based on camera or LiDAR) are some of the state-of-the-art target detection models based on a single sensor which achieves high accuracy results. Therefore, these models can provide further information regarding the state of our proposed model in comparison with the current state-of-the-art target detection models.

Finally, the influence of the lighting condition in the target detection task is discussed to prove the relevance of the sensor fusion. For this task, a reduced synthetic dataset has been created to generate night frames (500 frames) based on the original KITTI camera frames.

## 5. Results

In this section, a comparison of our proposed DNN and the state-of-the-art models for target detection will be presented for a deeper understanding of the advantages of our technique. The parameters that are compared in Table 2 are the size of the models in the second column, accuracy (for easy, medium and hard to detect targets according to the KITTI dataset) in the third column and latency in the fourth column. These parameters have been selected since they are the most relevant ones when executing target detection at the network edge taking into account the memory constraints of edge devices and latency requirements for autonomous vehicles.

Table 2 shows how the achieved general accuracy results when using our proposed Deep Fusion Network model for easy, medium and hard to detect targets (shown in the third column of Table 2 respectively for all the classes in the studied dataset) are lower than the rest of the studied models. In this comparison, the DGFN algorithm achieves the highest accuracy results for easy, medium and hard to detect targets. Nevertheless, the targets used during the training and testing of the DGFN and CLOCs algorithms were reduced subsets of the KITTI dataset. These subsets only include some of the classes instead of the whole list of targets. Consequently, this comparison must be understood as an estimation of the accuracy.

On the other hand, the model size of the proposed model is considerably smaller than the rest of the compared models. For example, it is 92.1% smaller than the DGFN model. The rest of the compared models do not provide information about their memory size so a direct comparison is not possible. Therefore, the model size can only be compared with the DFGN model. Nevertheless, the MV3D model [32], which preprocesses 3 inputs individually, as well as the CLOCs [27], which executes simultaneously a 3D and 2D detection, and MS-CNN [33], which studies independently each subset in the LiDAR point cloud of each target, can be assumed to require a larger memory due to their high complexity in comparison to our proposed MLSF model.

The memory reduction has been achieved due to the optimization of the model for the network edge by applying multiple techniques such as quantization [36] and pruning [37]. These techniques are further explained in Section 3.2. At the same time, the mobilenet detector integrated in the model, as well as the pruning of nonrelevant layers and parameters, leads to a faster execution as the previous table also shows.

As a conclusion from this table, it is possible to observe the tradeoff between the accuracy and memory/latency. Therefore, our algorithm has been designed following the network edge constraints in order to achieve a reduced latency and memory consumption. However, the accuracy results are 7.77%, 2.50% and 2.53% (easy, medium and hard to detect targets, respectively) smaller than the DGFN model. This leads to the conclusion that this model should be implemented in a collaborative approach with other networks to ensure high reliability for applications such as autonomous driving.

From this point on, now our proposed model will be compared with other algorithms for target detection which are not based on sensor fusion. In this case, since the goal of the comparison is to discuss the improvement of the robustness as well as the general accuracy of the models for all the studied classes, the comparison will be based on the used input data, the latency and the accuracy achieved by the model, as shown in Table 3.

It is possible to observe in Table 3 how our proposed model outperforms the SSD structure using a single data input. This can result from the lack of information when using a single sensor as previously discussed, which leads to a lack of robustness during the target detection. Overcoming this problem is one of the main reasons to apply sensor fusion techniques, as explained in [19]. In real scenarios such as the context of autonomous vehicles, the weather conditions can affect the data acquisition of each individual sensor (i.e., water on the camera lens after the rain, LiDAR laser absorption in fog conditions, etc.). When using a sensor fusion approach, this problem can be mitigated due to the bidirectional reinforcement of the data.

On the other hand, the Deep Manta model [23] outperforms our model as well as the SA-SSD [34] and MonoPair [35] when it comes to target detection accuracy. Regarding the latency, except the SSD structure, the rest of the studied models have a similar execution time to our model even when they study the data from a single sensor. Therefore, even when they achieve similar results of latency and accuracy, the robustness of the single sensor models is reduced in comparison with our MLSF model, following the criteria of [19].

Finally, the suitability of these models for edge devices must also be considered, since most of these models integrate complex layers such as 3D Convolutions which are not supported in edge devices like the Google Coral TPU. On the other hand, our MLSF has been designed following the layer restrictions of this device to ensure its correct function.

**Figure 7:**
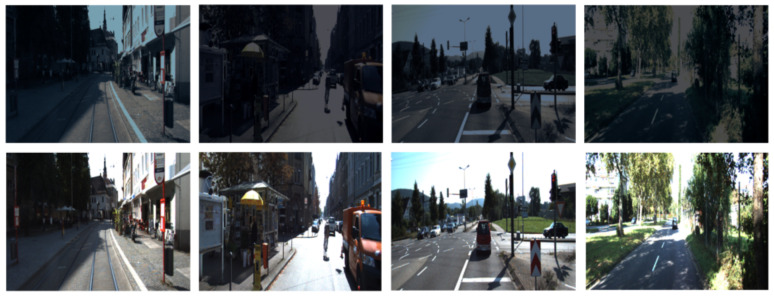
Synthetic night frames (**top** row) and original images (**bottom** row).

**Figure 8:**
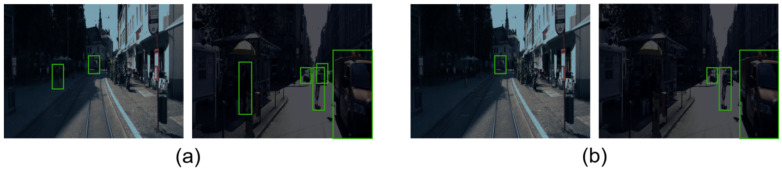
Target detection in night frame with (**a**) sensor fusion algorithm and (**b**) only camera.

The influence of the lighting conditions has been researched using some of the frames from the KITTI dataset and reducing their luminosity to generate synthetic night frames as shown in Figure 7. Due to the darkness of these images, the accuracy of the target detection in these frames when using a SSD mobilenet trained with the original KITTI dataset dropped to 73.81%–65.41%–39.63% (easy, medium and hard to detect targets) when using only the camera data. One possible reason is that in bad light conditions, the camera sensor may not be able to gather information about all the targets in the scenario. This problem can be observed in the top images of Figure 7 where it is hard to see the targets in comparison with the bottom images. However, when using our proposed sensor fusion algorithm, the model can detect targets that have not been located when using only the camera, as shown in Figure 8. LiDAR data is not affected by the poor light conditions due to the fact that it is based on measuring the time taken to receive the reflection of a transmitted laser pulse rather than measuring the external light reflected by the targets. The accuracy achieved with our model in these dark frames was 83.18%–80.02%–47.83%. Consequently, it is possible to observe how the LiDAR data reinforce the camera data by generating a map of relevant areas of the image, leading to a higher accuracy.

After these comparisons, it can be observed how our proposed model achieves high accuracy results for the researched task but it does not improve the state of the art. However, when taking into account the latency and memory constraints, our model achieves edge capabilities that are not present in the rest of the compared models while improving its robustness in comparison with single sensor models.

## 6. Conclusions

A Multi-Level Sensor Fusion deep neural network has been developed and tested in the Google Coral TPU Edge Device in this paper for target detection using camera and LiDAR sensors. The sensor fusion layers integrated in this model generate feature maps that are combined at multiple levels to produce a joint data representation that has been tested on the KITTI dataset.

The accuracy results do not surpass the state-of-the-art accuracy shown by other sensor fusion models such as DGFN [19]. Nevertheless, our model still achieves a 90.92%, 87.81% and 79.63% accuracy results for easy, medium and hard to detect targets in the challenging KITTI dataset while requiring a 92.1% less memory than the DGFN model [19]. As a result of the optimization applied to the proposed model, the latency of the model has also been reduced to 0.057 s, outperforming the rest of compared algorithms.

At the same time, the advantages of using a sensor fusion approach rather than a single sensor model have been studied by comparing our proposed algorithm with other single sensor target detection algorithms. This comparison is shown in Table 3 where it is possible to observe how some of the single sensor models achieve better accuracy than our proposed algorithm. However, we studied the effect of using only camera data for the target detection task and we showed how the accuracy drops to 73.81%–65.41%–39.63% (easy, medium and hard to detect targets) in cases where the light is not good. Since the LiDAR data does not depend on the light of the scenario, using a sensor fusion approach helps to achieve an accuracy of 83.18%–80.02%–47.83%. This proves that even if high accuracy can be achieved by a single sensor model, these algorithms are not robust when facing changes in the environment in comparison with a sensor fusion algorithm.

We can conclude that the proposed model has been designed in order to fit in edge devices as well as time constraints applications such as autonomous driving, taking into account a tradeoff among the accuracy, latency and memory size.

## Figures and Tables

**Table 1:** Parameters for the model training.

Parameter	Setting
Epochs	500
Batch size	6
Optimizer	SGD
Initial learning rate	$1\times 10^{-3}$
Final learning rate	$1\times 10^{-5}$
Momentum	0.9

**Table 2:** Comparison of LiDAR-Camera fusion networks for target detection.

Model	Size of the Model	Accuracy (%) Easy–Medium–Hard	Latency (s)
MLSF (proposal)	56.4 MB	90.92–87.81–79.63	0.057
DGFN [19]	713 MB	98.69–90.31–82.16	0.73
CLOCs [27]	–	88.94–80.67–77.15	0.1
MV3D [32]	–	95.01–87.59–79.90	0.36
MS-CNN [33]	–	93.98–89.92–79.69	0.5

**Table 3:** Comparison of of our model with no-sensor fusion algorithms.

Model	Data	Latency (s)	Accuracy (%) Easy–Medium–Hard
MLSF (proposal)	LiDAR-Camera	0.057	90.92–87.81–79.63
SSD [7]	Camera	0.003	87.14–84.37–75.74
SSD [7]	LiDAR	0.003	85.17–71.52–67.36
Deep Manta [23]	Camera	0.7	97.58–90.89–82.72
SA-SSD [34]	LiDAR	0.04	95.03–91.03–85.96
MonoPair [35]	Camera	0.06	96.61–93.55–83.55

## Data Availability

The dataset used during the explained research in this paper is public under the name KITTI dataset, which was developed by A. Geiger et al. [2].
